# PROTOCOL: Teacher professional development for disability inclusion in low‐ and middle‐income Asia‐Pacific countries: An evidence and gap map

**DOI:** 10.1002/cl2.1201

**Published:** 2021-11-09

**Authors:** Syeda K. Ahmed, David Jeffries, Anannya Chakraborty, Petra Lietz, Amit Kaushik, Budiarti Rahayu, David Armstrong, Kris Sundarsagar

**Affiliations:** ^1^ Australian Council for Educational Research Adelaide South Australia Australia; ^2^ Australian Council for Educational Research New Delhi India; ^3^ Australian Council for Educational Research Jakarta Indonesia; ^4^ RMIT University School of Education Bundoora Victoria Australia; ^5^ Australian Council for Educational Research Kuala Lumpur Malaysia

## Abstract

According to prior research, teacher readiness and capability are key contributors for successful transition towards disability inclusive education, yet in‐service teacher professional development for disability inclusion remains an under‐researched area. The key objective of this evidence and gap map (EGM) is to locate evidence on interventions for disability inclusion focused teacher professional development (TPD) in low‐to‐middle‐income‐countries (LMICs) in the Asia‐Pacific region. As such, it will illustrate different levels of evidence for TPD interventions as well as where there is no evidence (i.e., gaps). In other words, the EGM can make agencies aware where they might be operating in an area that is evidence‐free or evidence‐weak so they can take up interventions that are evidence‐based or collect evidence for the intervention they are presently supporting. Thus, the ultimate goal for the EGM is to assist funders and implementing agencies when making decisions as to how to support LMICs in the region to reach their aim of developing quality teachers for the global inclusive education agenda (target SDG 4.c).

## 1 BACKGROUND

### 1.1 The problem, condition or issue

The United Nations' 2030 Agenda for Sustainable Development calls for “inclusive and equitable quality education that promotes lifelong learning opportunities for all” (UNESCO, 2020, p. 1). In addition, the Sustainable Development Goal target 4.5 particularly focuses on inclusive education (IE) for the vulnerable and children with disabilities receive a strategic mention (UNESCO, 2016). According to General Comment No. 4 (Article 24) of the CRPD:

…some groups are more at risk of exclusion from education than others, such as: persons with intellectual disabilities or multiple disabilities, persons who are deafblind, persons with autism or persons with disabilities in humanitarian emergencies (CRPD, 2016, p. 3).

Advocates of educational inclusion call for a fundamental reform of schools and the modernisation of education systems (Azorín & Ainscow, 2020). An important clarification by UNICEF on how to implement inclusion in schools highlights the transformative role of inclusive education, “…making sure that teaching and the curriculum, school buildings, classrooms, play areas, transport and toilets are appropriate for all children at all levels”, thus emphasising that “inclusive education means all children learn together in the same schools” (UNICEF, 2017, p. 1). Similarly, UNESCO's “concept note” for the 2020 Global Education Monitoring (GEM) Report on Inclusion and Education indicates that the definition of inclusion has changed over the years from students with disabilities requiring separate classes and specialised teaching techniques to “a broader view, focused on ensuring that all students and students with disabilities are included in mainstream classes” (UNESCO, 2018, p. 4).

#### 1.1.1 Disability IE

Disability is a formal diagnostic label for the difficulties with everyday life faced by an individual (Armstrong & Squires, 2014) and has been defined as “a complex and multidimensional issue” (DFAT, 2016, p. 7). However, the focus is primarily on impairment, which captures the impact of a disability on the daily life of a student. An emphasis on impact rather than on diagnostic classification has been recommended by researchers as it relates to the supports and possible interventions necessary to facilitate inclusion (Armstrong & Squires, 2014).

Inclusion of students with disabilities has many advantages for all students, and “promotes cooperative, collaborative activities and increases positive attitudes towards disability, reducing stigma and discrimination and leading to inclusive societies” (DFAT, 2019, p. 4). Prior studies have noted significant benefits of IE for children with disabilities, particularly children with severe, complex, or multiple disabilities (Hunt, 2020; Katz & Mirenda, 2002). Studies have pointed out the advantages of IE for students with disabilities in terms of improved learning outcomes, including academic gains, improved communication and motor skills, higher social engagement (Hunt, 2019), stronger reading and mathematics skills, increased attendance rates, fewer behavioural problems, better social connections, and improved transition to postsecondary level (Hehir et al., 2016).

Research over the last two decades suggest how a range of factors operating at different levels affect the implementation of disability inclusion in educational settings. Thus, the implementation of policy initiatives at state or local level to promote social inclusion (Bills et al., 2020), school leaders' commitment to inclusion (Ainscow, 2020) as well as teacher practices in the classroom (Finkelstein et al., 2019), have emerged important factors. In addition, attitudinal barriers by teachers responsible for implementing disability inclusion have emerged as a reoccurring theme and found to important for the effective implementation of inclusion (Moberg & Savolainen, 2003; Savolainen et al., 2020; Van Mieghem et al., 2020).

These attitudinal barriers need to be looked at from a broader perspective. While teachers are an essential component of education systems, this is particularly the case in low‐to‐middle‐income‐countries (LMICs) where infrastructure and resources tend to be scarce, leading to additional challenges for disability inclusion into education settings (DFAT, 2019; UNESCO, 2020). More specifically, the GEM 2020 *Inclusion and Education* report describes barriers such as large pupil to teacher ratios, a lack of education support, weak professional teacher networks and a lack of autonomy over content (UNESCO, 2020).

Evidence from LMICs also suggest that teachers often lack the knowledge and skills for recognising and supporting students with disabilities (Ghimire, 2017; Kutcher et al., 2013; Shari & Vranda, 2015). Moreover, a lack of encouragement for teachers (e.g., a lack of increased pay or improved work conditions) (Muwana & Ostrosky, 2014) and widespread teacher‐centred methods of instruction (Arbeiter & Hartley, 2002) further impede the implementation of inclusion in these contexts (Wapling, 2016). Examples from Cambodia and India illustrate these issues where classroom practices were dependent on more traditional, less‐interactive teaching methods, in addition to overcrowded classrooms, scarce teaching resources and overambitious curricula, which made it harder for teachers to deliver one‐to‐one or small group teaching (Singal et al., 2018; Song, 2015).

#### 1.1.2 Issues affecting disability IE in the Asia‐Pacific region

In the Asia Pacific region, around one‐third of the children who are out‐of‐school have a disability (Modern et al., 2010) which indicates the need for appropriate education services that support the learning goals of children with disabilities to unleash their full potential (DFAT, 2015). Additionally, 52.7% of students with disabilities drop out of secondary schools, mostly from mainstream schools (UNESCAP, 2019). The 2015 data from 21 education systems in the Asia and Pacific region suggests that 19% of children (on average) with disabilities attended special primary schools (UN, 2018). Often, children with disabilities dropped out because of the financial burden on their families or contextual challenges (UN, 2018). One of Australia's key responses to this challenge has been through the provision of funds to “improve the accessibility to and quality of education for people with disabilities through policy dialogue, teacher training, curriculum development and education infrastructure” (DFAT, 2015, p. 10) in the region. Yet, the transition from segregated schooling to IE and teacher education reforms has been sluggish (Forlin, 2010; Wu‐Tien et al., 2008).

In Southeast Asia, teachers and preservice teachers mostly hold negative attitudes towards IE for students with disabilities (Forlin et al., 2007; Forlin et al., 2009; Sharma et al., 2006). Some reasons for this include a “lack of policy enforcement, lack of resources, lack of trained personnel, inflexible school system, merit‐oriented educational system, and also, societal attitude towards disability” (Bradshaw & Mundia, 2005, as cited in Low et al., 2018, p. 237). The influence of community/societal attitudes and beliefs on the beliefs and attitudes of teachers cannot be ignored. Collectively, studies by Hopf et al. (2017), Kuzma et al. (2016) and Kamenopoulou and Dukpa (2018), in Fiji, Papua New Guinea and Bhutan, respectively, highlight several attitudinal barriers to the effective implementation of disability inclusion in education in these LMICs.

Even in some high‐income countries in the region, such as Hong Kong and Singapore, high parenting pressure within some communities can lead parents to internalise social stigma (Mak & Kwok, 2010; Wong et al., 2015) which results in keeping their children with disabilities at home.

In most schools in this region, educational segregation of students with disabilities is widely accepted, and teachers largely believe it is appropriate for children with disabilities to be taught by special education teachers (Lee & Low, 2013; Low et al., 2018). In Malaysia, for instance, “it is expected that the preservice teachers in the regular subject areas would not perceive that it is their responsibility to teach students with disabilities, whilst the special education teachers would perceive teaching students with disabilities to be their distinct responsibilities” (Low et al., 2018, p. 238).

Besides, mainstream teachers may not be using teaching‐learning practices suitable for inclusive classrooms and “there is widespread acknowledgement that pedagogy is out of sync with the demands and challenges of the inclusive educational environment” (Rieser, 2013, p. 68). This is enhanced by the reality that teaching and learning in the Asia‐Pacific region is often driven by assessment results, creating a conflict between high achievement scores and inclusion (Forlin, 2010). Some mainstream teachers may even be pushing out students with disabilities from their classrooms because they are not sufficiently skilled to manage inclusive classrooms (Nes et al., 2017).

Also, research has shown that teachers require in‐depth training to learn how to effectively implement assistive technologies (Blossom Cygnet et al., 2019; McMillan & Renzaglia, 2014) that help students with disabilities to perform tasks and improve their functional capacity to participate in everyday activities.

Lately, this transition to disability inclusion has gained momentum in the region and it is widely acknowledged that funding effective teacher professional development programmes has the potential to create a profound impact on the wellbeing and school outcomes of students with disabilities. In this context, Australia is one of the key partners in supporting the education of students with disabilities by providing funds to the development of teacher training programmes in the region (DFAT, 2015).

Against this background, an evidence gap map (EGM) of teacher professional development (TPD) interventions supporting the inclusion of students with disabilities is useful and timely.

### 1.2 Scope of the EGM

TPD programmes are the key to transitioning to disability IE (CRPD, 2016). Since disabilities are complex, with changing definitions and thresholds for identification, teachers require regular professional learning to support disability inclusion (Forlin & Sin, 2010). One recent study from transnational and cross‐sector perspectives has suggested that to enable inclusion teachers “require professional learning that is collaborative, interprofessional, and acknowledges that the challenges they face are multifaceted.” (Beaton et al., 2021, p. 1). Although globally, IE is accepted as the most suitable approach to ensure universality and nondiscrimination in the right to education, many countries and especially resource poor LMICs, still have students with disabilities learning in a range of settings including special schools, integration classes in regular schools as well as in inclusive classrooms. To prevent this dilution of inclusion is the purpose of UNICEF's statement (2017) which is explicitly calls for special schools to cease as they are incompatible with inclusion.

This current EGM focuses on LMICs in the Asia‐Pacific region, covering 41 education systems as specified by the Australian Government Department of Foreign Affairs and Trade's (DFAT) (2018) list of economically developing countries. Many of these LMICs have education systems which need support in different areas including infrastructure, school governance reforms, teacher education, teacher recruitment and management, and learning assessment systems. Others are only starting their journey towards disability inclusion. Thus, for example, Fiji established the *2016 Policy on Special and Inclusive Education* which documents the need for preparing teachers for screening and referring students with disabilities (Ministry of Education Heritage & Arts, 2016; UNESCO, 2020), while in Gujarat, a state in western India, health and education departments collaboratively developed a training programme for the early identification of children with learning disorders such as dyslexia (Shastri, 2019; UNESCO, 2020). Some other countries are yet to establish policies which would result in the delivery of professional development opportunities for inclusion and supporting children with disabilities (UNESCO, 2020). For instance, in Bangladesh, teachers have reported an absence of professional development programmes (both pre‐ and in‐service) for supporting children with disabilities (Rahaman, 2017). International data from TALIS 2018 show that even with 52% of teachers in primary education, participating in TPD on teaching students with special needs in the 12 months before completing the survey, around 28% of teachers still reported a high need for it (OECD, 2021). Besides, the UNESCO GEM report notes a high demand from teachers in many countries for TPD programmes that support teaching children with disabilities (UNESCO, 2020).

While both pre‐ and in‐service teacher development programmes are needed to support teachers in transitioning to an IE system, the current EGM compiles information on *in‐service TPD interventions* only for various reasons.

- In‐service programmes can have a more immediate impact on the inclusion of students with disabilities in classrooms as they focus on practices and attitudes of current teachers.
- In‐service learning programmes are usually practice‐oriented with suggestions of how to make pedagogical practices more inclusive.
- Preservice education does not always equip teachers with competencies required to deal with everyday classroom challenges (Forlin, 2010). “Whether newly qualified teachers (NQTs) consider that they are sufficiently prepared to teach students with SEN in regular classes continues to be a cause for concern…” (Forlin, 2010, p. 180).
- Many teachers who have been in the profession for decades may not have received any formal training on disability IE. A study examining the skills of regular primary and secondary school teachers in Delhi in India found that nearly 70% of regular schoolteachers did not get training in special education and lacked experience of working with children with special needs (Das et al., 2013).

In summary, while most EGMs tend to have a broader scope, given the importance of the issue in this region, the authors are focused on synthesising evidence of TPD interventions for disability IE in the Asia‐Pacific LMICs only. This study and its scope have been supported by discussions with key funders and education experts in the region—such as DFAT and Australian Council *for* Educational Research (ACER) offices in India, Indonesia, and Malaysia—where stakeholders agreed on the need to have more information about the TPD interventions focused on disability inclusion in this region.

### 1.3 Conceptual framework of the EGM

Research shows that the provision of high‐quality IE is mainly influenced by teachers and their ability to support and acknowledge students' heterogeneous needs (Gomendio, 2017; Moen, 2008; Schwab & Alnahdi, 2020). More specifically, TPD is particularly relevant in the context of resource‐scarce LMICs in the Asia–Pacific region where teachers empowered with the right skills through interventions for the inclusion of students with disability can have significant impact on student outcomes (Chakraborty et al., 2019; UNESCO, 2017).

According to a model put forward by Finkelstein et al. (2019), inclusive teacher practice has five key aspects, namely instructional practice, organisational practice, socio/emotional practice, determining progress, and collaboration and teamwork. Teachers' expectations and beliefs‐in‐action resulting from social, cultural, and political influences have a dominating effect on teaching and learning in inclusive classrooms (Florian & Rouse, 2001; Howes et al., 2009). Thus, disability inclusive TPD not only needs to focus on eliminating stigma associated with disabilities but also create awareness and understanding of these issues to empower teachers.

In addition, it is equally important for education systems to assist teachers in developing the capabilities and confidence necessary to be inclusive of students with disabilities. In a high‐quality education system, teachers are supported through educational policies that focus on teachers' wellbeing and inclusion, preservice learning, and ongoing professional development (Darling‐Hammond & Cook‐Harvey, 2018).

**Figure 1:**
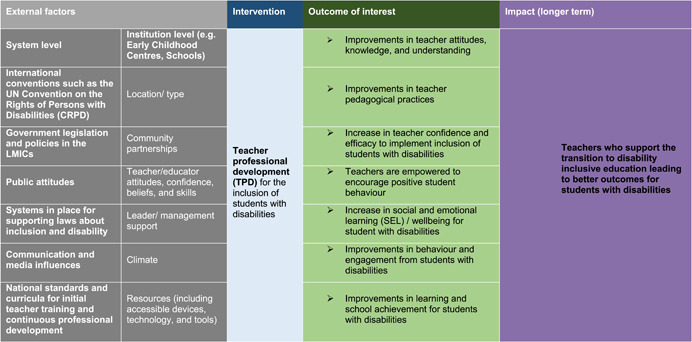
Conceptual framework of the EGM

Figure 1 provides a conceptual framework for exploring the disability inclusive TPD interventions and how these are linked to the outcomes of interest. This model does not represent a full theory of change of how specific interventions are meant to create impact. However, it does provide an overview of the relationships between external factors, interventions and outcomes and ultimate impact.

### 1.4 Why it is important to do this EGM

The Asia‐Pacific region is frequently affected by a range of natural disasters that impact the education of all children (UNESCAP, 2019) and that make it particularly difficult to provide quality education to children with disabilities when they occur (INEE, 2009). The current COVID pandemic has created additional obstacles to the transition to disability inclusion education in most LMICs (World Bank, 2020). The Christian Blind Mission (CBM) Australia for UNICEF's East Asia and the Pacific Regional Office and UNICEF Australia emphasises a further need to support teachers with training on disability inclusion, before schools re‐open and as schools establish “clear and adapted guidelines for social distancing and personal protection measures for staff supporting children with disabilities who may require additional personal care assistance requiring physical contact, such as getting around the school or using bathroom facilities” (UNICEF, 2020, p. 6) and provision of additional TPD, support, and mentoring for empowering teachers (UNICEF, 2020).

Therefore, a mapping of disability inclusive TPD interventions in this region is valuable and timely to gain more insights into the current situation and future needs for this sector. The content focus suggested for this EGM helps to keep this evidence synthesis manageable, appropriate, and relevant for interested funders and implementing agencies, who primarily support disability IE in the LMICs of the Asia‐Pacific region. The geographical focus means a greater potential for TPDs to be replicated or adapted as some countries in the region share several common cultures, backgrounds, and histories.

### 1.5 Existing EGMs and/or relevant systematic reviews

An earlier critical review by Waitoller and Artiles (2013) looked at research evidence from professional development studies focused on IE and found six types of TPD for IE: action research, on‐site training, university classes, professional development schools, online courses, and a special educator's weekly newsletter on how to include children with disabilities. However, this review could not locate any systematic review on TPD for IE and most reviews on TPD focused on studies conducted in Australia the UK, and the United States.

A recent meta review by Van Mieghem et al. (2020) identifies four substantive aspects of the implementation of IE: (1) attitudes towards IE; (2) teachers' professional development fostering IE; (3) practices enhancing IE and (4) participation of students with SEN. Van Mieghem and colleagues identified four reviews that highlights the TPD for inclusion theme: Kurniawati et al. (2014); Loreman (2014); Qi and Ha (2012); Roberts and Simpson (2016). A key finding in this area is that TPD is more effective when it focuses on specific student needs or disabilities, rather than on inclusion generally (Kurniawati et al., 2014), while a focus on specific teachers' concerns and their teaching context is the most helpful in encouraging change in teachers' practice (Kurniawati et al., 2014; Qi & Ha, 2012; Roberts & Simpson, 2016). Van Mieghem et al. (2020) concludes that TPD on evidence‐informed inclusive practices leading to successful teacher experiences is the cornerstone for the implementation of IE.

A current EGM on disability interventions (Saran et al., 2020) illustrates various initiatives for improving health, education, livelihood, social issues, empowerment and advocacy and governance for people with disabilities. However, this review reports only a single study on in‐service TPD in Kenya (Carew et al., 2019).

A key point to note is that most research in this space focuses on evidence from interventions that attempt to improve skills in the students with disabilities “rather than addressing institutional or environmental barriers, which are often the key focus of disability‐inclusive development” (Kuper et al., 2020, p. 2). For instance, an earlier review by Bakhshi et al. (2013) analysed programmes that increased the accessibility to education for children with disability aged between 4 and 18 years across economically developed and developing countries but did not include any TPD intervention.

A recent Rapid Evidence Assessment (REA) by Kuper et al. (2018) of *What Works to Improve Educational Outcomes for People with Disabilities in Low‐ and Middle‐Income Countries* focussed on interventions to improve educational outcomes for people with disabilities in LMICs, which reported a few TPD interventions (Carew et al., 2019; DeVries et al., 2018; Martin et al., 2001) from China, Kenya, and Uganda, respectively.

In summary, prior research identifies teacher readiness as a major factor for a successful transition towards disability IE while relevant work summarised here either does not cover TPD or cover interventions only from countries outside the Asia‐Pacific region. Hence, this EGM is timely and highly focused to provide a useful information base for targeted stakeholders.

## 2 OBJECTIVES

As researchers and policy makers are often unaware of the extent of the evidence base, an EGM is a way of making explicit and accessible different interventions on a certain topic in a specified geographic area, to “guide users to available relevant evidence to inform intervention and design and implementation” (White et al., 2020, p. 3).

The key objective of this EGM is to locate evidence on interventions for disability inclusion focused TPD in LMICs in the Asia‐Pacific region. As such, it will illustrate different levels of evidence for TPD interventions as well as where there is no evidence (i.e., gaps). In other words, the EGM can make agencies aware where they might be operating in an area that is evidence‐free or evidence‐weak so they can take up interventions that are evidence‐based or collect evidence for the intervention they are presently supporting (White et al., 2020).

Thus, the ultimate object for the EGM is to assist funders and implementing agencies when making decisions as to how to support LMICs in the region to reach their goal of developing quality teachers for the global IE agenda (target SDG 4.c), in addition to helping them attain the targets for SDG 4.1 (i.e., by 2030, ensure that all girls and boys complete free, equitable and quality primary and secondary education leading to relevant and effective learning outcomes) and SDG 4.5 (i.e., by 2030, eliminate gender disparities in education and ensure equal access to all levels of education and vocational training for the vulnerable, including persons with disabilities, indigenous peoples and children in vulnerable situations) (UNESCO, 2016).

## 3 METHODS

### 3.1 Defining EGMs

EGMs “are a systematic evidence synthesis product” (White et al., 2020, p. 1) intended to guide researchers and policymakers towards high quality evidence for identifying research gaps, informing research priority setting, and supporting evidence‐based decision making (Katz et al., 2003; Saran & White, 2018). Over time, different agencies have defined such evidence maps in different ways and used different approaches to generating such maps. However, Saran and White (2018) discuss key components that should be present in any definition of evidence maps (p. 9) which include the following:

- Systematic
- The type of evidence included
- The content of the map
- The structure of the map
- Graphical display
- Accompanying description of map
- Intended users.

Results from such evidence syntheses are valued by development partners who prefer to make investment decisions which are based on high quality evidence (e.g., DFAT, 2015a, 2015b; DFID, 2013; Jones, 2012; USAID, 2019). In recent years, such maps have gained popularity, particularly in the international development field. Thus, for example, a recent “map of maps” commissioned for international development interventions (Phillips et al., 2017) reported as many as 73 maps (Saran & White, 2018). While most evidence maps are broader in scope a few are quite focused (e.g., Bakrania et al., 2018; Robinson & Rust‐Smith, 2017).

**Figure 2:**
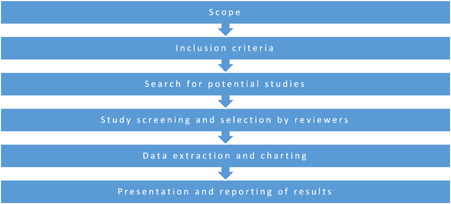
Steps for conducting an evidence and gap map (adapted from: Campbell Collaboration, n.d.; Saran & White, 2018)

Figure 2 outlines the process involved in conducting this EGM which is based on the methodological steps suggested by the Campbell Collaboration (White et al., 2020). This method involves (a) the development of the review's scope, (b) the setting of inclusion criteria, (c) searching for and identifying relevant studies, (d) screening and assessing studies for inclusion, (e) extracting and charting the data and (f) presenting and reporting the results.

In line with the Campbell EGM guidance that critical appraisal of all included studies is desirable but not mandatory (Saran & White, 2018; White et al., 2020), a decision was made in the current EGM to exclude this step as the timeframe for this study is shorter than a full‐sized systematic review. The search for this EGM is quite comprehensive and systematic, comparable to a systematic review search, however, some of the more stringent search steps will not be taken to ensure this study is completed within the planned timeframe. For example, the search statement relies heavily on subject terms to provide a more specific search with more relevant results, while the search statement for a systematic review would have been broadened to rely less on subject terms and to consider more variations including proximity operators.

The data extraction step will essentially follow the elements suggested by Saran and White (2018, p. 16) by charting the:

- Intervention categories
- Outcome categories
- Status of the study: completed or ongoing
- Geographical coverage of the study, where applicable
- Inclusion criteria of any included systematic reviews
- Primary study design.

The visual representation of the results is intended to be published as an EGM through the ACER data visualisation website (currently under construction, similar to 3ie's platform, see e.g.: https://egmopenaccess.3ieimpact.org/evidence-maps/improving-young-childrens-learning-economically-developing-countries-scoping-review).

A brief report will also be produced as part of this EGM which will discuss the extent of evidence and its characteristics, such as geographical distribution and the study designs.

### 3.2 EGM framework

**Figure 3:**
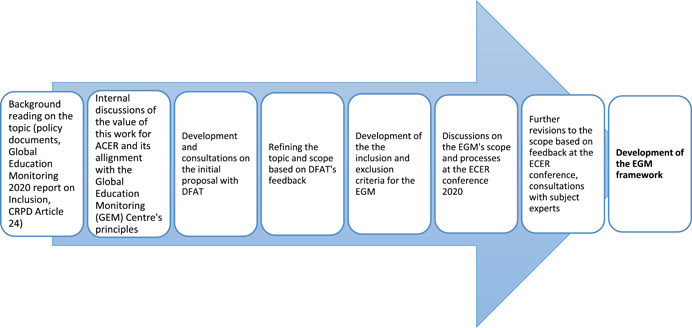
Process for the development of the EGM's intervention/outcome framework

Figure 3 illustrates the process for the development of the EGM's intervention/outcome framework which has been guided by the main objective for this study.

Table 1 shows that, based on the conceptual framework previously (see Figure 1), this EGM has two main dimensions, with the type of TPD interventions in rows and intended outcomes in columns. As can be seen, interventions are categorised either in terms of disability types, including physical, mental, developmental, sensory, as well as multiple or complex needs or in terms of special interest groups such as learning difficulties, specialised tools, approaches, and techniques for support for students with disabilities. The outcomes of TPD interventions are further categorised depending on whether those outcomes are mainly aimed at teachers or students. Each of these two groups has further sub‐categories. For teachers, TPD outcomes may focus on their attitudes, knowledge and understanding, pedagogical changes, enabling positive student behaviour or teachers' confidence and efficacy to implement inclusion. When a TPD includes some student outcomes these may be focused on students' overall learning and achievement, classroom behaviour, and engagement as well as their social and emotional learning and wellbeing.

**Table 1:** The proposed intervention‐outcome dimensions for the EGM

TPD Intervention	Intended teacher outcomes				Intended student outcomes		
	Attitudes, knowledge, and understanding	Pedagogical changes	Enabling positive student behaviour	Confidence and efficacy to implement inclusion	Learning and achievement	Behaviour and engagement	Social and emotional learning/wellbeing
*Disabilities and impairments:*							
Physical							
Mental Health							
Developmental							
Sensory							
Multiple or complex needs							
Special interest groups:							
Learning difficulties							
Specialised tools, approaches and techniques							

#### 3.2.1 TPD Intervention categories

Based on formal diagnostic categorisations some groupings are suggested below to distinguish between the different types of disabilities or impairments for the purpose of categorising the intervention focus on this EGM:

- A *physical impairment* affects the mobility or physical capacity of individuals. It may result, for example, from acquired brain injury, spinal cord injury, Spina bifida, Cerebral Palsy, and/or Epilepsy (Aruma, 2019a).
- The World Network of Users and Survivors of Psychiatry suggested a change in the way persons with mental health disabilities are described and are to be referred to as persons with psychosocial disabilities (WNUSP, 2008). While we acknowledge the term psychosocial disability, for the purposes of this EGM *mental health condition* or another recognised classification such as *developmental disability (DD)* will be used.
- The American Psychiatric Association lists conditions such as Schizophrenia, Obsessive‐Compulsive, and Related Disorders as *mental health condition* (APA, 2020).
- *Developmental disabilities (DDs)* are defined by Zablotsky et al. (2019) as “a group of lifelong conditions due to an impairment in physical, learning, language, or behaviour areas” and notes “Children diagnosed with developmental disabilities typically require services to address behavioural and developmental challenges” (p. 144). While persons with ASD and Intellectual disability (ID) carry increased risk of developing a mental health issue (Matson & Williams, 2013) these are distinct, and therefore ASD and ID can be classified as a developmental disability (Zablotsky et al., 2019).
- A *sensory impairment*, on the other hand is associated with impediments to the senses, such as sight, hearing, smell, touch, and taste (Aruma, 2019b). DSM‐5 categorises communication disorders as a component of sensory disabilities comprising of Language Disorder, Speech Sound Disorder, Childhood‐Onset Fluency Disorder (Stuttering), and Social (Pragmatic) Communication Disorder (Paul, 2013). The American Speech‐Language‐Hearing Association (ASHA) also recognises hearing disorders as a communication disorder (ASHA, 1993).
- A more complex form of disability is when an individual has *multiple impairments and complex needs* that is, when two or more conditions simultaneously impact a person's ability to live their life independently. There could be any combination of disabilities impacting someone, for instance a sensory and a physical impairment which causes unique learning needs that cannot be accommodated in a special education setting designed for a specific disorder (AIHW, 2009). There could also be increased complexities from negative attitudes, stereotyping or prejudice by others.

Another way of grouping interventions will be using special interest groups, for example the EGM will cover interventions which support particular *learning difficulties*, such as, difficulties in learning to read (dyslexia), and write (dysgraphia) or other areas of learning, such as mathematics (dyscalculia), or interventions that teach/train teachers in *specialised tools, approaches and techniques* (e.g., functional behavioural assessment, cognitive strategy instruction, collaborative inquiry and/or use of individual learning plans).

#### 3.2.2 Outcome categories

As the EGM is focussed on TPD, for interventions to be included must have at least one of the following outcomes aimed at teachers:

- Attitudes, knowledge and understanding
- Pedagogical practices
- Enabling positive student behaviour
- Confidence and efficacy to implement inclusion.

In addition, interventions may also have intended student level outcomes such as:

- Learning and achievement
- Behaviour and engagement
- Social and emotional learning/wellbeing.

These are discussed in detail in Table SA (see Supporting Information Appendix S1).

The online EGM will use the intervention‐outcome dimensions shown in Table 1 with circles of varying sizes in each cell to indicate the amount of available evidence (i.e., # of studies). For included systematic reviews, the size of the circles will be proportional to the number of studies that are included in the reviews or maps.

The EGM will also include functionality to enable studies and reviews to be filtered by country through a drop‐down menu. More information about the included studies will be available by hovering over the circles, such as:

- The total number of interventions included
- First author's name and year of study publication
- Country where the intervention was conducted.

An interactive geographical map will also be generated that indicates evidence availability in each LMIC in the Asia‐Pacific region. By clicking on each of the countries where evidence is available the following information will appear around the included studies and reviews.

- Title of the study/systematic review
- Author information
- Publication year
- Link to the study/review
- Status of the intervention (i.e., ongoing, completed)
- Study design/method
- Funding/implementing agency (if available) particularly for practice‐based interventions.

### 3.3 Criteria for including and excluding studies

The criteria detailed in Table 2 will be considered when deciding eligibility to include or exclude a study/review in this EGM.

**Table 2:** Inclusion and exclusion criteria for the EGM

Selection criteria	Inclusion	Exclusion
Publication year	Studies published between 2000 and 2021	Studies published before 2000
Publication status	Completed and on‐going	Planned
Study design	Primary studies (including quantitative, qualitative, or mixed methods), and systematic reviews and EGMs that are focussed on TPD for disability inclusion	Reviews or EGMs that focus on TPD but are not focused on TPD for inclusion and disability
		Reviews or EGMs that include TPD studies for disability inclusion from countries that are not listed under Asia and Pacific on the DFAT (2018) list of developing countries
Publication language	Studies/reviews published in English only	Studies published in a language other than English
Population	In‐service teacher professional development (TPD) and/or professional learning programmes	Interventions for preservice teachers during initial teacher education
Interventions	Programmes that support teachers to understand the needs of students with disabilities	Programmes that focus only on supporting teachers to accommodate other diverse groups, such as ethnic groups, migrant communities, children belonging to low‐socioeconomic status, refugees, and other minority groups
	Programmes that support the integration and inclusion of students with disability in mainstream classrooms	
	Programmes in special school settings that support students with disabilities	
	Evidence for practice‐based interventions (i.e., initiatives that have been undertaken/are being undertaken in LMICs in the region of interest) where there is sufficient information available about these in the grey literature searched	Practice‐based interventions (i.e., initiatives that have been undertaken/are being undertaken in LMICs in the region of interest) without sufficient information about the TPD programme (or TPD component)
	Details should at least include:	For example:
	Intervention (or component) name that focuses on disability inclusive TPD Intervention categories Outcome categories Status of the programme Geographical coverage Funding agency/implementing agency	Statements that are broad and vague, without providing details about a programme (e.g., XYZ programme has been running in the Pacific Islands and has supported students with disabilities through several initiatives, that also includes teacher professional training)
Context (geographic location and settings)	Interventions in low and middle‐income countries (LMICs) in the Asia Pacific region	Interventions in high‐income countries (HIs) in the Asia Pacific region or countries (including LMICs) from a different region.
	A relevant study found in a review which is from a country of interest will be included as a primary study—if the review covers interventions conducted in other regions and countries, and therefore cannot be included as a review based on this inclusion criteria.	
	Interventions in early childhood settings including nurseries, playgroups, child‐care centres, or preschools; and school settings including, K‐12 mainstream schools and/or special education schools.	Interventions for teachers who are beyond school levels (such as faculties at tertiary education level institutions or vocational institutes).
Intended outcomes	At least one teacher outcome must be reported. Details are specified in the EGM outcomes framework (see Table 1; also see Table SA, Supporting Information Appendix S1).	None
Quality	Not to be restricted based on any quality assessment.	None

#### 3.3.1 Types of study designs

Since the main purpose of this review is to map the evidence for in‐service TPD for disability inclusion in classrooms, a wide variety of study designs will be accepted if they add information on the topic of interest and help to identify evidence gaps.

This review will therefore consider both qualitative and quantitative (e.g., experimental, quasi‐experimental, before and after studies without control groups, descriptive studies) (see Table 2 for more details). The studies could follow any of these research methods or follow a mixed methods design if they meet the inclusion criteria. Additionally, any study with a TPD programme impact summary/description which provides insights into the inclusion of students with disabilities for in‐service teachers in LMICs in the Asia‐Pacific region may be eligible for inclusion if it meets all the criteria.

Any systematic review/and or EGMs focusing solely on TPD for disability inclusion with studies from LMICs in the Asia Pacific region only will also be eligible for inclusion.

#### 3.3.2 Status of studies

The EGM will cover both completed and on‐going studies which are presently in‐progress and have some form of evidence documented and available.

However, ongoing studies found through such systematic searches, which are past their registration cut‐off date or with uncertainty about their completion, or without sufficient details will not be included.

#### 3.3.3 Types of intervention

Any type of teacher professional development/learning programme/intervention or in‐service training opportunity with the aim of creating disability inclusive classrooms for students with physical, mental, developmental, sensory, and or multiple or complex needs will be eligible. Also, any TPD focused on supporting learning difficulties and supporting teachers to use specialised tools, approaches and techniques will be included (see Section 3.2 for more details).

For reviews in which only a subset of the interventions is eligible for inclusion in the map, only the relevant interventions (i.e., the relevant primary studies) will be included in the data extraction and mapping.

The included interventions will cover strategies to support disability inclusion related outcomes in classrooms.

#### 3.3.4 Types of population

Practicing teachers or special needs educators in early childhood centres or child‐care services, preschools, and schools who are working with children/students between the ages of 0–18 years.

The review also includes teachers and educators who work with students with special needs in mainstream schools or special schools or special education classrooms in mainstream schools.

#### 3.3.5 EGM framework outcomes

##### 3.3.5.1 Intended

An intervention must have a teacher outcome and may also report student outcomes.

Teacher outcomes

- Attitudes, knowledge and understanding
- Pedagogical practices
- Enabling positive student behaviour
- Confidence and efficacy to implement inclusion.

Student outcomes

- Learning and achievement
- Behaviour and engagement
- Social and emotional learning/wellbeing.

See Section 3.2 and Supporting Information Appendix S1 for more details.

##### 3.3.5.2 Unintended

Any potentially adverse or unintended outcomes of the interventions will also be noted in the EGM report for the final studies included in the EGM.

#### 3.3.6 Other eligibility criteria

##### 3.3.6.1 Types of Location/Situations

Studies which explore interventions in LMICs in the Asia Pacific region will be included. The reason for this geographical focus is due to the Asia‐Pacific region being an area of strategic interest for many development partners (DPs) who value evidence and gap maps when making key investment/funding decisions (e.g., DFAT, 2015a, 2015b; DFID, 2013; Jones, 2012; USAID, 2019).

##### 3.3.6.2 Types of settings

The intervention could be set in any of the following:

- Early childhood settings including nurseries, playgroups, child‐care centres, or preschools
- School settings including, K‐12 mainstream schools and/or special education schools.

### 3.4 Search methods and sources

An initial limited search of development partner portals was undertaken to scope several potentially relevant papers, including previous literature reviews and systematic reviews on in‐service teacher training for inclusion of students with disabilities in LMICs. Results of these searches has been used to further develop the EGM's search terms.

A broad range of bibliographic databases and repositories will be electronically searched to help develop the search strategy. The search platforms include:

- A+Education
- British Education Index
- Education Research Complete
- ERIC
- SCOPUS
- 3ie Development Evidence Portal (Evidence Hub)
- Campbell Collaborations Systematic Reviews and EGMs portal (Better evidence for a better world)
- EPPI (UCL‐UK) Database of Educational Research
- Teacher Reference Centre (EBSCO)
- Google scholar.

However, ongoing studies found through such systematic searches, which are past their registration cut‐off date or with uncertainty about their completion, or without sufficient details will not be included.

A sample search statement has been provided (see Supporting Information Appendix S2). The search statement relies heavily on subject terms to provide a more specific search with more relevant results, while the search statement for a systematic review would have been broadened to rely less on subject terms and to consider more variations including proximity operators, for this EGM some of the more stringent search steps will not be taken to ensure this study is completed within the planned timeframe.

The search for unpublished studies—and practice‐based interventions—will be conducted through Development Partner Publication portals such as UNICEF, World Bank, USAID, the Australian DFAT and the UK's Foreign, Commonwealth and Development Office (formerly DFID). Potential papers will be sought through “snowballing” as a result of searching bibliographies and reference lists of papers located during the search process, as well as specific searches of relevant grey literature. Potential on‐going interventions that are identified through any of the above‐mentioned sources will also be screened for inclusion in the EGM.

The EGM will clearly distinguish where evidence is practice‐based or emerging from ongoing interventions that are selected from grey literature and match the inclusion criteria. For example, the following grey literature sources will be searched to look for any evidence that can be included in this EGM:

- Programme/Project websites: DFAT (2019) Disability Inclusive Education in Fiji: Learning from the Australian Aid funded Access to Quality Education Program (AQEP)—This programme's Disability Inclusion Strategy facilitated many positive outcomes including increased enrolment and attendance of children with disabilities, increased skills and confidence amongst teachers and several policy and system level changes.
- University research websites: Monash University (2016) Pacific Indicators for Inclusive Education (Pacific‐INDIE): Case Studies—There are four case studies presented from Fiji, Vanuatu, Samoa and the Solomon Islands—the four key countries involved in the development of the Pacific INDIE final set of indicators. Each of the four countries have made varying progress towards IE and face their unique contextual challenges. Common challenges across all four countries include the translation of policy to practice, the need for ongoing advocacy and the need for training of teaching staff.

The search will be rerun by the review team close to publication of the EGM by Campbell Collaboration if the initial search date is more than six months from the planned publication date. The additional results will be thoroughly screened for potentially eligible interventions/studies. The research team will fully incorporate any new interventions/studies identified in the search rerun if this can be accomplished within the proposed timeframe for publication.

### 3.5 Data extraction, coding and management

#### 3.5.1 Screening and study selection

##### 3.5.1.1 Title and abstract screening

All search records will be screened against inclusion and exclusion criteria. During this first round of screening, two reviewers will independently look at the titles and abstracts and only those deemed relevant to the topic will make it to the next round of full‐text screening.

##### 3.5.1.2 Full text screening

The full text for the studies which will be included from the title and abstract screening stage will also be screened against inclusion and exclusion criteria. At the end of this stage, only studies which are expected to be included in the EGM will remain and data will be extracted and charted from these.

The entire search process and the screening outcomes will be documented using a PRISMA Flow Diagram (Moher et al., 2009) so that the readers should be able to follow, and potentially replicate, all steps of the review process (see Supporting Information Appendix S3).

#### 3.5.2 Data extraction and presentation

The data extraction process will involve gathering information about:

- The study title, year, author(s)
- The aim, brief description, content, and length of each intervention/study
- The setting (early childhood, mainstream school, or special school) and country
- Target population and sample size
- The intended professional development outcomes
- The research outcomes of the intervention (and information about programme effectiveness if any.
  For systematic reviews the following information will be extracted:
- The review title, year, author(s)
- The purpose and methods
- The number of studies included, and key themes analysed
- The intended outcomes and/or any effectiveness data (such as effect size) reported.

At least two reviewers will independently extract data from each study and resolve any differences through consultations. This will involve in‐depth discussion of the study and the inclusion/exclusion criteria until an agreement is reached. Any contextual information about the reason for an intervention or descriptive information about how it had achieved its effects will also be recorded. The entire data extraction process will be managed using MS Excel. A template for data extraction is provided in Supporting Information Appendix S4.

## 4 ANALYSIS AND PRESENTATION

### 4.1 Unit of analyses

For this EGM, each study about an intervention will be considered as the unit of analysis for primary studies. Therefore, if multiple studies report on the same intervention all of the individual studies will be included as separate pieces of evidence.

Besides, having the intervention rather than the study as the unit of analysis is problematic as different study designs will address different questions about an intervention. In addition, as mentioned earlier, no quality appraisal of studies will be undertaken. Therefore, it will not be possible to decide which study reporting on an intervention would be better to include over another.

### 4.2 Presentation

Findings from the EGM will be presented in two ways, namely a cost‐free and publicly accessible online EGM and an accompanying report.

The EGM is intended to be an online representation of Table 1. In each cell, a circle will show whether evidence is available for a certain intervention/outcome intersection. In addition, the size of the circle will reflect the amount of available evidence, with the size increasing as the amount of evidence increases. Hovering over and clicking on a circle will enable easy access to the underlying evidence/references. The EGM will be developed using common web development languages (e.g., HTML, CSS, and JavaScript).

Depending on the evidence found as a result of the study, the EGM may also include filters which can be applied to select interventions in terms of additional characteristics, such as:

- Location of the interventions (i.e., LMICs with evidence)
- Setting type (i.e., early childhood, mainstream or special schools).
- Length of intervention
- Delivery mode (e.g., face‐to‐face, online)
  The accompanying EGM report will be developed in line with the structure suggested by the Campbell Collaboration. This accompanying EGM report, will provide:
- A synthesis of the findings of the EGM
- An in‐depth discussion of particular areas of interest (e.g., countries with more evidence; evidence gaps; the prevalence of evidence by subregions—South Asia, Pacific, East Asia; the prevalence of evidence by service setting etc.)
- Observations about potential implications for policy, practice, and research
- A plain language statement of EGM findings.

## 5 ADDITIONAL NOTES

### 5.1 Stakeholder engagement

Advice on an earlier version of the EGM proposal from DFAT and CBM has contributed to refining the direction of this study. In addition to first scans of evidence emerging from initial topical searches, feedback from the following stakeholder engagements has further clarified the topic and scope of this EGM:

- Initial consultations with the GEM Centre Executives of the value of this study for ACER and its alignment with the GEM Centre's principles.
- Sharing of the initial proposal with DFAT Education Section and their Disability Technical Partners Christian Blind Mission (CBM) Global Disability Inclusion Group during December 2019.
- Guidance on the scope and inclusion/exclusion criteria from subject experts—Dr David Armstrong, Editor, Journal of Research in Special Educational Needs (JORSEN) and Dr Jane Jarvis, Cochair, Research in Inclusive & Specialised Education (RISE), Flinders University.
- Presentation of the scope, methods, and initial findings at the *Educational Research (Re) connecting Communities* (ECER) 2020, online conference (in the Network 4: Inclusive Education forum), organised by the European Educational Research Association (EERA) during August 2020.

## CONTRIBUTIONS OF AUTHORS

Working closely with the GEM Centre, this EGM is being undertaken by a team from the Australian Council for Educational Research (ACER) led by Ms. Syeda Kashfee Ahmed. Ahmed has been trained through The Centre for Evidence‐based Practice South Australia (CEPSA): A Joanna Briggs Institute Centre of Excellence. She has worked extensively in the field of education and has contributed to papers in teacher professional learning and development. Some recent relevant reports include: Ahmed et al. (2020) and Dix et al. (2019).

The core review team also includes Dr. David Jeffries, Ms. Anannya Chakraborty and Dr. Petra Lietz. Dr. Lietz was a coauthor of several systematic reviews (Best et al., 2013; Lietz et al., 2017) and meta‐analyses (Lietz 2006a, 2006b) demonstrating her expertise with these methods.

Two of the authors, Ahmed and Lietz, have recently completed a scoping review for the GEM centre on young children's learning in economically developing countries (Jackson et al., 2019).

The core team members will be primarily responsible for the key review tasks, including eligibility screening, quality assessment, coding of studies, data extraction, presentation and writing of the review report.

The review team also includes Mr. Amit Kaushik (ACER India), who has recently contributed to a related thematic review on assessments for students with disabilities in the Asia‐Pacific region along with Ms. Chakraborty (Chakraborty et al., 2019). Other research team members include Ms. Budiarti Rahayu (ACER Indonesia), Dr David Armstrong (RMIT University) and Ms. Kris Sundarsagar (ACER Malaysia). Dr Armstrong has worked extensively in the field and is currently a special education and IE lecturer, editor of the Journal of Research in Special Educational Needs (JORSEN), and provides expert advice to Amnesty International, Parliamentary Inquiries and other key stakeholders about enabling educational inclusion and reducing exclusion. All team members will provide expert knowledge, particularly on regional issues regarding in‐service TPD, and assist the core team members to identify relevant evidence for the EGM.

The review team is also supported by Ms. Jenny Trevitt, Senior Librarian (Information Dissemination) and ACER's literature search specialist with more than ten years' experience as a librarian in ACER's Cunningham Library. Ms. Trevitt has also been directly involved with information retrieval for previous systematic reviews. The team is further supported by Mr Toby Carslake (ACER, Adelaide) who will be developing the online interactive EGMs for graphically presenting the results of this review.

## DECLARATIONS OF INTEREST

The authors declare no conflict of interest.

## PRELIMINARY TIMEFRAMES

**Table:** 

Time period	Deliverables
November 2021	Protocol and literature search completed
December 2021	Study inclusion completed
February 2022	Draft EGM submitted
April 2022	Final EGM submitted

## PLANS FOR UPDATING THE EGM

Ms. Ahmed will be responsible for updating this EGM every five years, subject to funding availability from the GEM Centre.

## Supporting information

Supporting information.

Supplementary Information
