# Supplementary material for: PROTOCOL: Teacher professional development for disability inclusion in low‐ and middle‐income Asia‐Pacific countries: An evidence and gap map
Source: Campbell Syst Rev. 2021 Nov 9;17(4):e1201. doi: 10.1002/cl2.1201 (PMC8988775; doi:10.1002/cl2.1201)
Supplement: Supplementary file 2 — Supplementary Information [file CL2-17-e1201-s002.docx]

# Appendices

## Appendix 1. Outcome categories for the EGM framework

The proposed outcome categories for the EGM framework are featured in Table A. The table defines each of the intended outcome categories and provides examples for studies that will be included in the proposed EGM.

Table A. Outcome Categories for the EGM framework

| **Intended Outcomes** | **Description** | **Examples** |
| --- | --- | --- |
| Teachers |  |  |
| Attitudes, knowledge, and understanding of inclusion and disabilities | Relates to teachers’ attitudes (acceptance, willingness) towards students with disabilities and knowledge about and understanding of inclusive practices | *Friendly education to persons with disabilities.* A program which provided training for teachers to improve their understanding about persons with disabilities.  Salim, A., Hidayatullah, M. F., & Nugraheni, P. P. (2019). Investigating Effectiveness of Disability Friendly Education Training Modules in Indonesian Schools. International Journal of Education and Practice, 7(3), 286-293. |
| Pedagogical changes | Focuses on teachers’ gaining skills to improve pedagogical practice for the purpose of improving inclusive practices and educational outcomes for students with disabilities | *Transforming Thai Preschool Teachers' Knowledge on Inclusive Practice:* *A Collaborative Inquiry.* A paper which focused on effective teaching techniques for students with disabilities.  Agbenyega, J. S., & Klibthong, S. (2015). Transforming Thai preschool teachers' knowledge on inclusive practice: A collaborative inquiry. Australian Journal of Teacher Education, 40(7), 5. |
| Enabling positive student behaviour | Enables teachers to develop strategies to manage behaviour issues more effectively among students with disabilities | *Early Identification of Common Child Mental Health Problems.* Focused on training teachers on behaviour management.  Hussein, S. A., & Vostanis, P. (2013). Teacher training intervention for early identification of common child mental health problems in Pakistan. Emotional and Behavioural Difficulties, 18(3), 284-296. |
| Confidence and efficacy to implement inclusion | Relates to teachers’ confidence, and self- efficacy for implementing disability inclusive approaches / strategies | *Research conducted for the Kabataang Aralin sa Lahat Ibahagi (KASALI) Project:* A study of whether and how children with disabilities are being included in classrooms and early childhood centers. *S*ave the Children. (2018).  ECCD and elementary school teachers’ testimonies revealed that they benefited from KASALI project. It aided teachers in applying IE instructional strategies efficiently, including teacher’s awareness of cultivating learner self-esteem, and greater confidence in one’s capacity. |
| Students with disabilities |  |  |
| Learning and achievement | Results in positive learning and achievement outcomes for the students | *Professional Learning Program for Enhancing the Competency of Students with Special Needs.* Trained teachers to support children with ASD, and learning disabilities which led to improvements in students’ reading, spelling and mathematics.  Kantavong, P., & Sivabaedya, S. (2010). A Professional Learning Program for Enhancing the Competency of Students with Special Needs. International Journal of Whole Schooling, 6(1), 53-62. |
| Behaviour and engagement | Changes in students’ behaviour and engagement | *Cognitive Strategy Instruction on Deaf Learners.* A program which trained teachers and led to improvements in students’ behavioural outcomes and attention.  Martin, D. S., Craft, A., & Sheng, Z. N. (2001). The impact of cognitive strategy instruction on deaf learners: An international comparative study. American Annals of the Deaf, 146(4), 366-378. |
| Social and emotional learning/wellbeing | Changes to the way students thinks of/ feels about themself and of others (their peers), leading to changes in self-esteem, learning capacity and the sense of school-belonging | *Inclusion in Vietnam: More than a Quarter Century of Implementation.* Participating in this inclusive education training led to students exploring new ideas via cooperative learning among students, positive changes classmate attitudes and feelings about children with disabilities, an increased perception that all children should be valued members of a classroom.  Hai, N. X., Villa, R. A., Van Tac, L., Thousand, J. S., & Muc, P. M. (2020). Inclusion in VietNam: More than a quarter century of implementation. *International Electronic Journal of Elementary Education, 12*(3), 257-264. DOI: 10.26822/iejee.2020358219 |

## Appendix 2. Sample search statement

**SEARCH STATEMENT - ERIC (VIA EBSCO) SEARCH**

**15 July 2020**

Limiters - Date Published: 20000101-20201231

Search modes - Boolean/Phrase

Note:

- GE = LOCATION IDENTIFIERS
- SU=SUBJECT HEADINGS FROM THE THESAURUS
- Terms not proceeded by a field code were searched as a key word so that the search was not limited to any particular field. The keyword search included title and abstract searching.

COPY OF FINAL SEARCH STATEMENT

( GE (Asia OR “Pacific Islands” OR Afghanistan OR Bangladesh OR Bhutan OR Cambodia OR China OR India OR Indonesia OR Kazakhstan OR Korea OR Korea OR Kyrgyzstan OR Lao OR Malaysia OR Maldives OR Mongolia OR Myanmar OR Nepal OR Pakistan OR Philippines OR “Sri Lanka” OR Tajikistan OR Thailand OR “Timor Leste” OR Turkmenistan OR Uzbekistan OR Vietnam OR Cook Islands OR Fiji OR Kiribati OR “Marshall Islands” OR Micronesia OR Nauru OR Niue OR Palau OR Papua OR Samoa OR “Solomon Islands” OR Tokelau OR Tonga OR Tuvalu OR Vanuatu OR Wallis OR Futuna) OR “Pacific Island” OR “Pacific Islands” OR “Asia Pacific” ) AND ( SU Inclusion OR exclus* OR equit* OR inequit* OR SU “Equal Education” OR marginali* OR SU Disadvantaged OR "activity limitation" OR "participation restriction" OR SU "Special Education" OR SU "Special Needs" OR SU "Slow learners" OR "Differently abled" OR retard* OR SU Disabilities OR SU Disability OR SU Disorder* OR SU Impairments OR handicap* OR abnormal* OR SU Discrimination OR harass* OR SU Bullying OR “Student Diversity” OR SU “Mental Health” OR SU “Mental Disorders” OR SU Accessibility OR SU Poverty OR SU “Physical Development” OR SU sensory OR SU “Intellectual Development” OR psychosocial OR hearing OR SU Deafness OR SU Vision OR SU Blindness OR SU Delayed Speech OR SU Developmental OR SU Autism OR ASD OR SU “Asperger Syndrome” OR SU “Learning Problems” ) AND ( SU (“Early Childhood Education” OR “Child Care” OR Caregiver* OR School* OR “Elementary Education” OR “Primary Education” OR “Secondary Education” OR “Elementary Secondary Education” OR “Preschool Education” OR “Kindergarten” OR “Grade 1” OR “Grade 2” OR “Grade 3” OR “Grade 4” OR “Grade 5” OR “Grade 6” OR “Grade 7” OR “Grade 8” OR “Grade 9” OR “Grade 10” OR “Grade 11” OR “Grade 12” OR “Intermediate Grades” OR “Special Education”) OR “K-12” OR “K to 12” ) AND ( SU ("Professional development" OR training OR “teacher education”) )

275 RESULTS

## Appendix 3. PRISMA flowchart template

Records identified through database searching

Additional records identified through other sources

Records excluded during screening

- No professional learning intervention for in-service teachers
- Discussion/ policy papers/ reviews/ book chapters without mention of any intervention
- Studies on teacher perceptions / attitudes on inclusion / SEN
- Professional learning with no focus on inclusion or special education
- Study from high income countries or outside the Asia-Pacific region

Records after duplicates removed

Records screened (title and abstract)

Full-text articles assessed for eligibility

Full-text articles excluded, with reasons

Studies included in EGM

Figure C. PRISMA 2009 Flow Chart Template

Adapted from: Moher et al., 2009

## Appendix 4. Data extraction template

Table D. Template for data extraction from studies

| **Publication details** | | | **TPD Intervention details** | | | | | | | | **Study design and data collection** | | | **Outcomes** | | |
| --- | --- | --- | --- | --- | --- | --- | --- | --- | --- | --- | --- | --- | --- | --- | --- | --- |
| Key | Authors | Year | Theme | Country | Setting | Target population | Length | Brief description | Content | Study design | | Sample size | Intended | | Outcomes/ Effectiveness data (if available) | Unintended |
|  |  |  |  |  |  |  |  |  |  |  | |  |  | |  |  |
|  |  |  |  |  |  |  |  |  |  |  | |  |  | |  |  |
